# Treatment and Prognosis of Anaplastic Thyroid Carcinoma: A Clinical Study of 50 Cases

**DOI:** 10.1371/journal.pone.0164840

**Published:** 2016-10-19

**Authors:** Tian-Run Liu, Zhi-Wen Xiao, Hai-Neng Xu, Zhen Long, Fan-Qin Wei, Shi-Min Zhuang, Xiao-Mei Sun, Liang-En Xie, Jia-Sheng Mu, An-Kui Yang, Guan-Ping Zhang, Yi Fan

**Affiliations:** 1 Department of Otorhinolaryngology-Head and Neck Surgery, Sixth Affiliated Hospital of Sun Yat-sen University, Guangzhou, China; 2 Department of Radiation Oncology, University of Pennsylvania Perelman School of Medicine, Philadelphia, United States of America; 3 Department of General Surgery, Xinhua Hospital, affiliated to School of Medicine, Shanghai Jiao Tong University, Shanghai, China; 4 Institute of Biliary Tract Disease, Shanghai Jiao Tong University School of Medicine, Shanghai, China; 5 State Key Laboratory of Oncology in South China, Guangzhou, China; 6 Department of Head and Neck Surgery, Sun Yat-sen University Cancer Center, Guangzhou, China; West China Second Hospital, Sichuan University, CHINA

## Abstract

**Introduction:**

Although anaplastic thyroid carcinoma (ATC) is rare, it is one of the most aggressive human cancers. The optimal multimodal therapy policy of ATC is still debated, and a standardized treatment strategy remains to be established. This study aimed to evaluate the management aspect and prognosis of ATC.

**Materials and Methods:**

The data were analyzed retrospectively for 50 patients with ATC to evaluate the clinical characters, management and factors influencing survival. Survival analysis was performed by Kaplan-Merier method and log-rank test, and multivariate analysis was performed using Cox proportional hazard model.

**Results:**

The 1-year and 2-year overall survival rates (OS) were 48.0% and 26.0% respectively in all patients, with the 2-year OS of 40.0% and 31.0% and 6.3% for stage IVA, IVB and IVC respectively (*P* <0.05). In stage IVA and IVB patients, combined surgery with radiotherapy improved overall survival, and the 2-year OS were 50.0% and 35.7% respectively in the group with combined surgery with radiotherapy and the group with surgery with only (*P* <0.05). Postoperative radiotherapy improved local control rate in stage IVA and IVB patients (*P* <0.05). However, surgery, radiotherapy or chemotherapy could not improve the survival of stage IVC patients. Multivariate analysis showed that distant metastases, surgery, radiotherapy and tumor residue could predict the prognosis.

**Conclusion:**

Combined surgery and radiotherapy could improve overall survival in stage IVA and IVB patients. Patients with ATC have a bad prognosis. Distant metastases, surgery, radiotherapy and tumor residue are the most important factors affecting the prognosis.

## Introduction

Anaplastic thyroid carcinoma (ATC) accounts for 1–2% of all thyroid malignancies with an annual incidence of about one to two cases per million [1, 2]. Although ATC is rare, it is one of the most aggressive human cancers, and it causes up to 40% of deaths from thyroid cancer. The average survival time of ATC is only 6 to 8 months and the 5-year survival rate of ATC is only 0 ~ 10% [1, 3].

Despite different treatment approaches, ATC grows rapidly, invades adjacent tissues, and most patients die due to uncontrolled local tumor invasion or distant metastases. The treatment options for ATC include surgery, chemotherapy and radiotherapy. Some studies showed that multimodal therapy combining surgery, chemotherapy and radiotherapy, might achieve better results in avoiding death from local invasion and improving survival in some patients; however, ATC has an extremely low cure rate even with the very best treatments, and treatment of ATC is mostly palliative [4–6]. Surgical resection with adjuvant radiation therapy and chemotherapy may prolong survival or improve quality of life, however strong evidence is needed to support this conclusion [7, 8]. The optimal multimodal therapy policy is still debated and a standardized treatment strategy remains to be established. Furthermore, the rare incidence of ATC and its aggressive nature make it difficult to compare the outcomes of different treatments, especially in studies with small cohorts [4, 5].

In this study, we retrospectively reported the clinical and pathologic outcome of a scale of 50 ATC patients treated in our institution, in order to evaluate the best treatment strategy and the factors affecting prognosis.

## Patients and Methods

### Patients

Fifty ATC patients were treated in our hospital between January 1990 and December 2006, accounting for 2.55% of the patients with thyroid malignant tumor (50/1963) during the same period, including 26 males and 24 females with a male to female ratio of 1.08:1. The age ranged from 36 to 85 years old, with a median age of 60 years. The medical histories ranged from 10 days to 24 months.

The main clinical manifestations included: neck mass in 38 cases, hoarseness in 9 cases, difficulty breathing in 9 cases, difficulty swallowing in 8 cases and neck pain in 13 cases. In all the patients, 35 cases were involved in unilateral thyroid, 15 cases in bilateral thyroid and 21 cases had enlarged cervical lymph nodes (21/50, 42.0%) with the most common area was ipsilateral Level IV. Sixteen cases had distant metastases on their initial hospitalization (16/50, 32.0%), including lung metastasis in 12 cases, ipsilateral radius metastasis in 1 case and multiple bilateral rib metastasis in 3 cases. According to the standard of TNM staging (UICC, 2010), 15 cases were Stage IVA, 19 cases were Stage IVa and 16 cases were Stage IVC. Written Ethics Approval and Patient Consent from the Hospital Research Ethics Committee of Sun Yat-sen University Cancer Center and Sixth Hospital were obtained, and participants provided their written informed content to participate this study.

### Treatment

Treatment of the tumor and thyroid: for patients with tumor limited in unilateral thyroid lobe, total thyroidectomy was performed; while thyroidectomy with extensive resection of the surrounding tissues was performed for patients with tumor involved the surrounding tissues. Treatment of cervical lymph nodes: for patients with clinical or pathological cervical lymph nodes, level II to VI neck dissection was performed; while for patients with clinical negative cervical lymph nodes, level VI neck dissection was performed. Patients just underwent tracheotomy and tumor biopsy when they suffered from a wide range of tumor, severely invaded trachea (narrow diameter < 0.5 cm), or poor health status. Surgical margins are classified by the pathologist as R0 and R1 others; R0 indicates that no cancerous cells seen microscopically, while R1 means that cancerous cells can be seen microscopically; however the others includes tracheotomy, biopsy or non-surgical treatment.

Postoperative radiotherapy with a dose of more than 40 Gy is recommended in stage IVA and IVB patients and palliative doses should also be used to improve quality of life in some patients with widespread disease.

### Follow-up

Follow-up information was obtained by the telephone and outpatient review. Follow-up time started from the date of the first treatment and ended by December 15, 2015.

### Statistical Methods

SPSS 19.0 software was used for statistical analysis, counting data by chi-square test. Survival analysis was performed by Kaplan-Merier method, comparison among/between groups was performed using log-rank test, and multivariate analysis was carried out using Cox proportional hazard model. *P*<0.05 was considered statistically significant.

## Results

### Treatment results

In all these cases, 38 cases accepted total or extensive thyroidectomy with neck dissection in 16 cases (17 sides), and 3 cases accepted palliative resection of cervical lymph node, and 3 cases just underwent tracheotomy and tumor biopsy (Tables 1 and 2). The primary tumors were confined in the thyroid gland in 10 cases (10/50, 25.64%), while the tumor invaded out of the thyroid capsule in 29 cases (29/50, 74.36%). Microscopic positive surgical margin was found in 21 cases (R1) after radical surgery. The invaded surrounding structures included anterior cervical muscle and/or sternocleidomastoid in 21 cases, recurrent laryngeal nerve in 12 cases, trachea in 14 cases, esophagus in 10 cases, larynx in 3 cases, internal jugular vein or common carotid artery in 17 cases, jugular vein angle and clavicle in 2 cases, anterior superior mediastinum in 3 cases. Cervical lymph node metastasis was found in 21 cases (21/50, 42.0%), with ipsilateral Level IV as the most common involved area (11/19, 57.9%).

**Table 1:** Clinical characteristics of the 50 ATC patients.

Factor	Cases	Overall survival		*P* value
		1-year	2-year	
**Gender**				
Female	24	45.8	16.7	0.254
Male	26	50.0	34.6	
**Age**				
≤ 60 years	26	53.8	30.8	0.261
> 60 years	24	41.7	20.8	
**Diameter of primary tumor**				
≤ 4 cm	17	47.1	29.4	0.808
> 4 cm	33	48.5	24.2	
**T stage**				
T4a	19	63.2	36.8	0.125
T4b	31	38.7	19.4	
**Strap muscle invasion**				
No	28	50.0	35.7	0.462
Yes	22	45.5	13.6	
**Recurrent laryngeal nerve invasion**				
No	41	46.3	29.3	0.488
Yes	9	55.6	11.1	
**Trachea invasion**				
No	30	56.7	33.3	0.192
Yes	20	35.0	15.0	
**Esophageal invasion**				
No	40	50.0	27.5	0.255
Yes	10	40.0	20.0	
**Lymph node metastasis**				
No	29	48.3	34.5	0.187
Yes	21	47.6	14.3	
**Distant metastasis**				
No	34	58.8	35.3	0.006
Yes	16	25.0	6.3	
**Stage**				
VIA	15	60.0	40.0	0.021
VIB	19	57.9	31.6	
VIC	16	25.0	6.3	
**Surgery**				
R0	17	76.5	47.1	0.000
R1	21	47.6	23.8	
Other	12	8.3	0.0	
**Radiotherapy**				
No	34	35.3	17.6	0.004
Yes	16	75.0	43.8	
**Chemotherapy**				
No	42	47.6	26.2	0.270
Yes	8	50.0	25.0	
**Tumor residue**				
No	21	76.2	52.4	0.000
Yes	29	27.6	6.9	

**Table 2:** Treatment methods of the 50 ATC patients.

Treatment group	Case(s)
**Surgery only**	21
**Surgery plus radiotherapy or chemotherapy**	17
**Chemoradiotherapy**	1
**Chemotherapy only**	2
**Tracheotomy, biopsy or other**	9

Sixteen patients underwent radiotherapy including postoperative (external beam) radiotherapy in 15 cases, with the dose varying from 30 to 70 Gy, including more than 40 Gy in 15 cases. And 8 cases received chemotherapy, including postoperative adjuvant chemotherapy in 5 cases and palliative chemotherapy in 3 cases (Tables 1 and 2). The chemotherapeutic agents were mainly MAID (ifosfamide, adriamycin, and dacarbazine) for 2 ~ 5 courses.

Twenty-nine cases had tumor residual after their first treatments, including 6 cases just underwent tracheotomy and tumor biopsy without further treatments.

### Survival conditions

**Fig 1:**
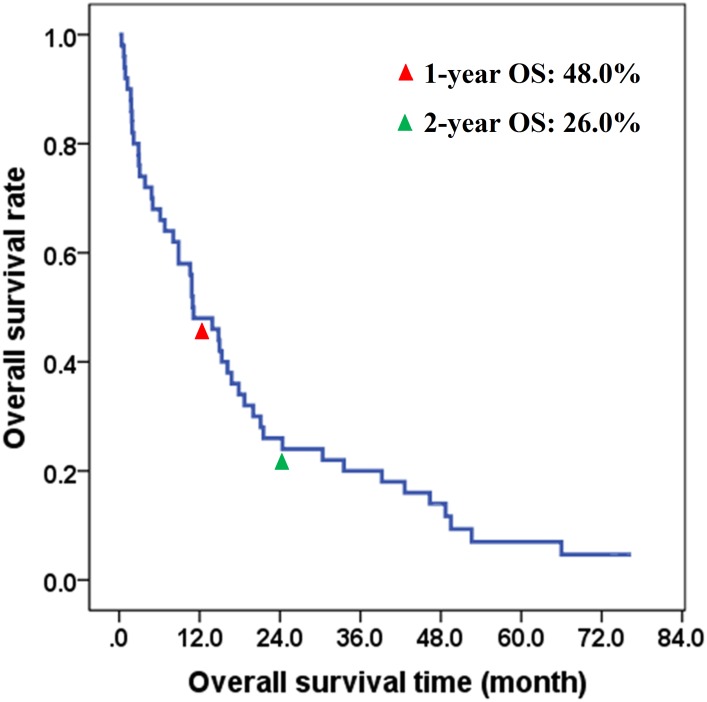
Overall survival of 50 ATC Patients.

By the latest follow-up on December 15, 2015, 3 cases were still alive and 47 cases died from cancer-related diseases, including 35 cases died of primary tumor, and 12 cases died from distant metastases. The 1-year, 2-year and 5-year overall survival rates / cancer-specific survival rates were 48.0%, 26.0% and 4.7% respectively in all patients, with the 2-year overall survival / cancer-specific survival rates of 40.0%, 31.0% and 6.3% for stage IVA, IVB and IVC respectively (*P* <0.05). The survival curve was demonstrated in Fig 1.

### Local control rate

After the first treatment, 29 patients survived with tumor (29/50, 56.0%), and the other 21 patients survived without tumor (21/50, 42.0%). The 1-year and 2-year local control rates were 26.0% (13/50) and 16.0% (8/50), respectively.

**Fig 2:**
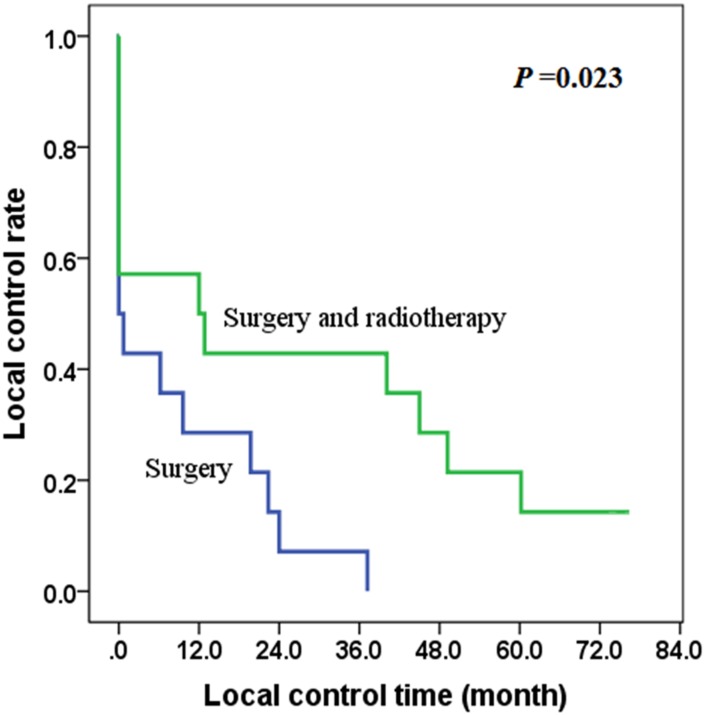
The effect of radiotherapy on local control of stage IVA and IVB patients.

In patients without distant metastasis (IVA and IVB), most cases were treated mainly with surgery (S) or combined surgery with postoperative radiotherapy (SR), and there was no significant difference in the R0/R1 ratio between the S group (7/7, 50.0%) and the SR group (5/9, 55.6%) (*χ*^2^ = 0.583, *P* = 0.704). The local control rate of the SR group was better than that of the S group (*χ*^2^ = 5.139, *P* = 0.023), with the 2-year local control rates of 42.9% and 7.1% respectively in the SR group and S group (Fig 2).

### The role of radical surgery on the survival of patients with ATC

**Fig 3:**
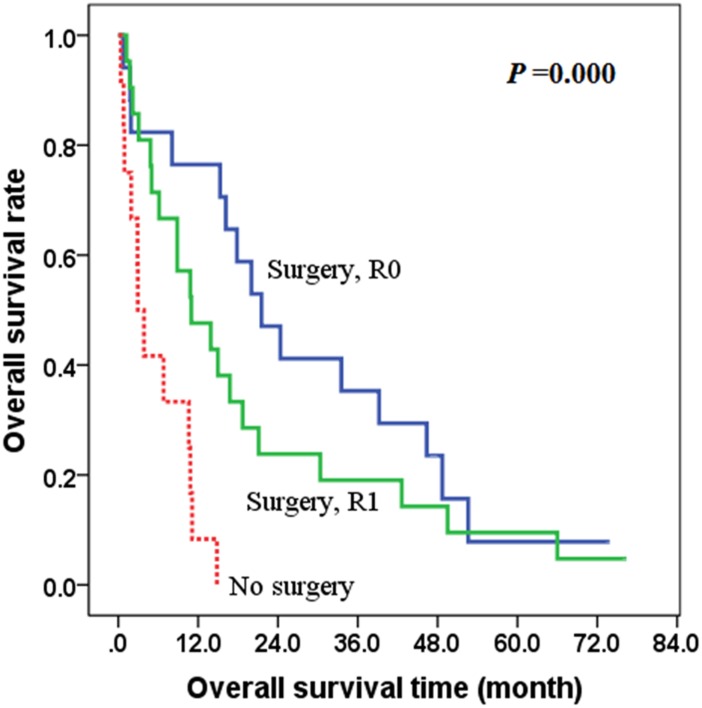
The effect of radical surgery on overall survival of ATC patients.

In all patients, radical (extensive) surgical treatment was performed in 38 cases. For surgical margin, 17 cases were R0 and 21 cases were R1. The overall survival of the R0 group or R1 group was better than that of the group without surgery (*χ*^2^ = 17.364, *P* = 0.000), with the 2-year overall survival rates of 58.3%, 31.3% and 0.0% respectively in the R0 group, R1 group and the group without radical surgery (Fig 3).

### The role of postoperative radiotherapy on the survival of stage IVA and IVB patients

**Fig 4:**
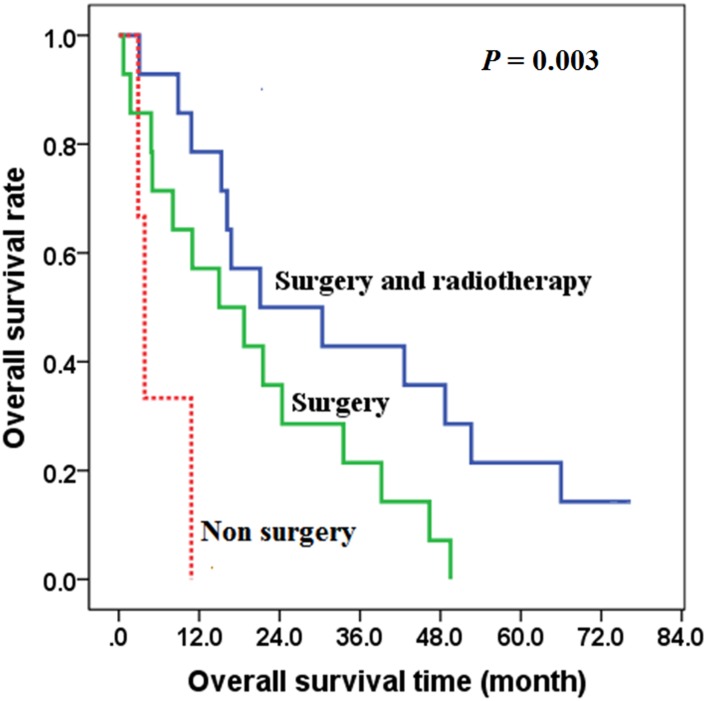
The effects of postoperative radiotherapy on overall survival of stage IVA and IVB patients.

In patients without distant metastasis (IVA and IVB), the overall survival of the group treated with combined surgery and postoperative radiotherapy was better than the group treated with only surgery (*χ*^2^ = 4.584, *P* = 0.032), with the 2-year overall survival rates of 50.0% and 35.7% respectively in the postoperative radiotherapy group and surgery only group (Fig 4). There was no significant difference in the R0/R1 ratio between the group treated with surgery only (7/7, 50.0%) and the group with postoperative radiotherapy (5/9, 55.6%) (*χ*^2^ = 0.583, *P* = 0.704).

The survival of patients with non-surgical treatment was worse than the postoperative radiotherapy group or surgery group (*χ*^2^ = 11.493, *P* = 0.003) (Table 3).

**Table 3:** The overall survival of different treatment groups of stage IVA and IVB patients.

Factor	Cases	Overall survival		*P* value
		1-year	2-year	
**Surgery+ radiotherapy**	14	78.6	50.0	
**Surgery only**	14	57.1	35.7	0.003*
**Radiotherapy or chemotherapy**	3	0.0	0.0	

* Surgery with radiotherapy vs Surgery only: *P* = 0.032.

### The role of different treatment methods on the survival of stage IVC patients

**Fig 5:**
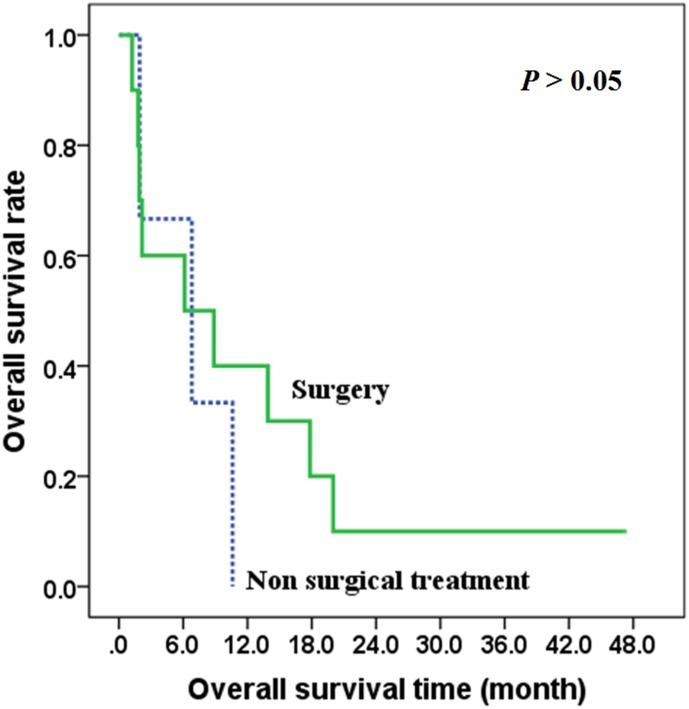
The effects of surgery on overall survival of stage IVC patients.

**Fig 6:**
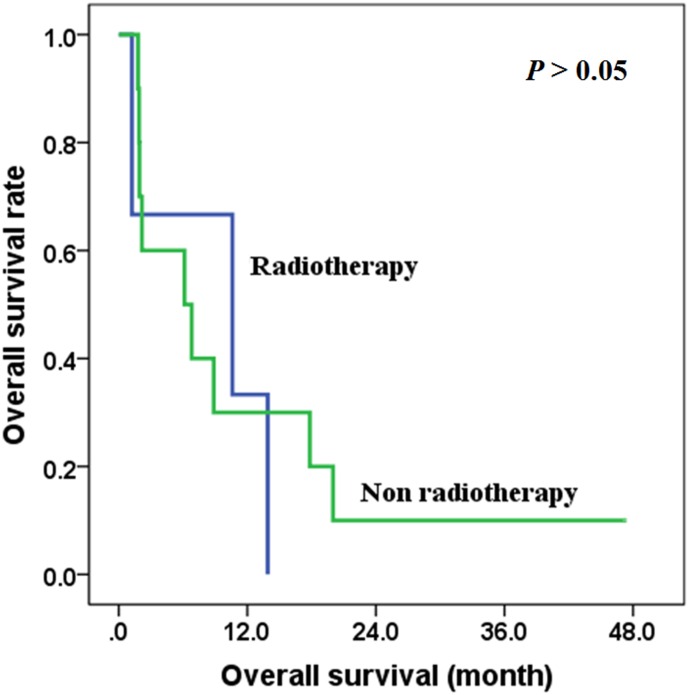
The effects of radiotherapy on overall survival of stage IVC patients.

**Fig 7:**
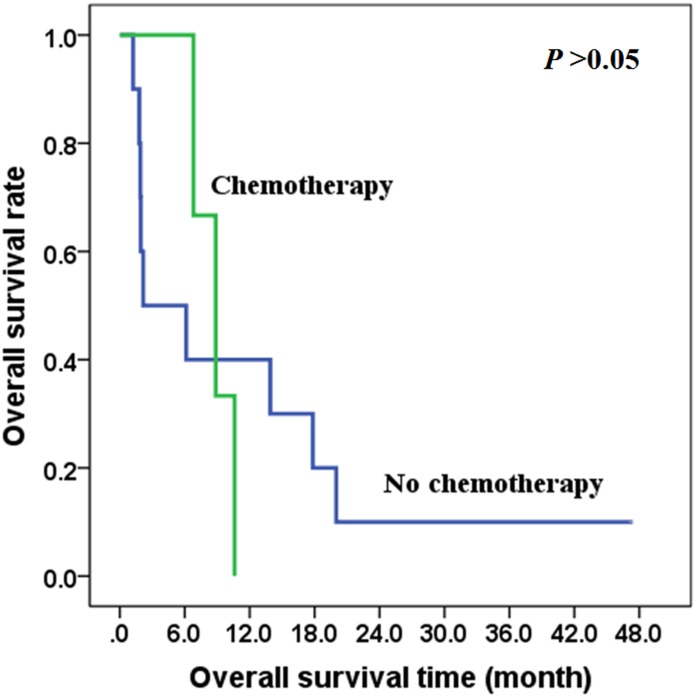
The effects of chemotherapy on overall survival of stage IVC patients.

In patients with distant metastasis (IVC), there was no difference between the overall survival time of those treated with surgery and those with non-surgical treatment (*χ*^2^ = 0.413, *P* = 0.521), with the 2-year overall survival rates of 50.0% and 35.7% respectively in the two groups (Fig 5). Also, there was no difference between the overall survival time of those treated with radiotherapy and those treated without radiotherapy (*χ*^2^ = 0.125, *P* = 0.724) (Fig 6), and there was no difference between the overall survival time of those treated with chemotherapy and those treated without chemotherapy (*χ*^2^ = 0.041, *P* = 0.839) (Fig 7).

### Multivariate analyses of the prognostic factors on overall survival

In order to evaluate the important factors affecting survival of patients, the possible factors that may affect prognosis, such as gender, age (> 60 years old, ≤60 years old), diameter of primary tumor, tumor confined to the thyroid gland or not, lymph node metastasis, trachea invasion, esophageal invasion, distant metastasis, surgical treatment, radiotherapy, chemotherapy and tumor residue were analyzed by multivariate Cox regression analysis. The results showed that the independent factors affecting the prognosis of patients underwent treatment were primary tumor size (diameter) less than 4 cm, distant metastases, surgery, radiotherapy, chemotherapy and tumor residue (Table 4). Multivariate analysis showed that distant metastases, surgery, radiotherapy and tumor residue could predict the prognosis.

**Table 4:** Multivariate analysis results of factors affecting the prognosis.

Factors	B	SE	Wald	*P*	Exp(B)
**Age**	-.282	.404	.486	.486	.754
**Diameter of primary tumor ≤ 4 cm**	-1.333	.407	10.718	.001	.264
**Limited to gland**	-.485	.466	1.081	.299	.616
**Strap muscle invasion**	.350	.419	.699	.403	1.419
**Trachea invasion**	.254	.432	.345	.557	1.289
**Esophageal invasion**	.181	.448	.162	.687	1.198
**Lymph node metastasis**	-.231	.362	.406	.524	.794
**Distant metastasis**	1.235	.397	9.678	.002	3.438
**Surgery**	-1.104	.532	4.306	.038	.331
**Radiotherapy**	-1.215	.448	7.350	.007	.297
**Chemotherapy**	-1.757	.587	8.972	.003	.173
**Tumor residue**	2.575	.590	19.022	.000	13.131

## Discussion

ATC accounts for 1% ~ 2% of all thyroid tumors, with the characteristics of fast progression, strong local invasion, high distant metastasis rate and so on. The tumor has a very poor prognosis, and the average survival time is approximately 6 to 8 months [1, 3, 7, 9]. Multimodal therapy combining surgery, chemotherapy and radiation therapy, might achieve better results in improving survival in some patients; however, ATC has a very low cure rate even with the extremely radical treatments [3, 4].

Compared with differentiated thyroid cancer, one of the notable characteristics of ATC is the aggressive invasion of the thyroid capsule and the adjacent structures, and early neck lymph nodes metastasis and distant metastasis [10]. In this study, 31 of the 50 cases (62.0%) with tumor invasion through the thyroid capsule, and the most common invaded surrounding structures included the strap muscle, recurrent laryngeal nerve, trachea and esophagus. The tumor was hardly removed completely in patients with extensive tumor invasion. Thirty-eight patients underwent extensive thyroidectomy, but local microscopic tumor residue was found in 21 cases. Tumor residue was a main factor affecting prognosis; therefore early screen of cancer and taking active measures in initial treatment, such as multimodal treatment, may help to improve prognosis [5]. ATC patients have a high regional lymph node metastasis rate [11], such as 42.0% in this group, with level IV as the most common area. Additionally, ATC patients have a high distant metastasis rate, such as 32.0% in this group with the lung as the most common metastatic site.

The main treatment strategies of ATC are surgery, radiotherapy chemotherapy and biotherapy [4–7, 12], and radical surgical treatment is still a key therapeutic method affecting the prognosis. This study showed that there was significant difference in survival rates among the radical surgical treatment group and non-surgical treatment group (*P* = 0.000). Some scholars found that survival outcomes were significantly higher in patients with resectable tumors than in those with unresectable tumors [2, 7, 10, 13]. Sugitani’s study showed that the 1-year survival rate was found to be significantly higher in resectable tumors than that in the unresectable tumors (39.0% and 10.0%, respectively; *P* < 0.0001), which showed that the important role of surgical treatment in ATC [13]. R0 (negative surgical margin), and R1 (gross resection, positive microscopic margin) might result in substantial improvement in local control and survival; however, most of these studies are biased because they are retrospective and not randomized to control for bias factors such as extent of disease or adjuvant treatments [14, 15].

This study also showed that postoperative radiotherapy can improve OS in stage IVA and IVB patients (*P* = 0.032), and the 2-year overall survival rates of 50.0% and 35.7% respectively in the postoperative radiotherapy group and the surgery only group. Also, our study indicated that postoperative radiotherapy (more than 40 Gy) improved local control rate in stage IVA and IVB patients (*P* = 0.023). Furthermore, multivariable analysis showed that radiotherapy is an independent factor affecting prognosis. Some scholars also suggested that postoperative radiotherapy might be an effective way in the treatment of ATC [5, 16]. A study from Glaser SM showed that high-dose radiotherapy (>59.4 Gy) resulted in improved survival in ATC, and radiotherapy is a prognostic factor [17]. Therefore, we recommend postoperative radiotherapy with a dose of more than 40 Gy in stage IVA and IVB patients and palliative doses should also be used to improve quality of life in some patients with widespread disease.

Chemotherapy drugs, including cisplatin, doxorubicin, vincristine, were used to treat ATC patients commonly; however, it is still controversial whether chemotherapy can prolong the survival time and improve prognosis [18, 19]. Theoretically, chemotherapy can control the small metastatic sites around the main primary tumor, reduce the tumor dissemination or increase the tumor resection rate by shrinking the tumor, or improve the effect of radiotherapy and improve the long-term curative effect. Nevertheless, it is lacked of strong evidence to prove the above opinions [18, 19]. Our study also showed that chemotherapy might be an independent factor affecting prognosis in multivariate analysis; however, this study is limited in chemotherapy cases by sample size, and this opinion still needs to be confirmed by extending the sample. A study from Haigh confirmed postoperative chemoradiotherapy can improve the survival rate [2]. Busnard B thought preoperative chemoradiotherapy can improve tumor resection rate and improve prognosis [20], however, Mclve found it didn’t significantly prolong survival time in patients who accepted a comprehensive treatment based on surgery and chemoradiotherapy [21]. Prospective studies with enlarged sample size are needed to confirm whether comprehensive treatment is better than simply surgical treatment.

The prognosis of ATC patient is very poor, and this study showed that the 1-year and 2-year overall survival rates were 48.0% and 26.0%, respectively in all patients. This study also indicated that the independent factors affecting the prognosis of patients underwent treatment were primary tumor size (diameter) less than 4 cm, distant metastases, surgical treatment, radiotherapy and tumor residue. Some studies showed that white blood cell count and whether to accept surgery plus postoperative radiotherapy were the independent factors influencing the prognosis of ATC [22]. Lo C evaluated the factors influencing the prognosis of ATC and found that age may be associated with prognosis, limited lesion means a better prognosis, while with or without differentiated thyroid cancer may have nothing to do with the prognosis; the size of primary tumor was associated with prognosis, and the tumor resection rate was higher when the tumor diameter was less than 5 to 6 cm, which leads to a good prognosis [23]. Therefore, age and tumor size may also be prognostic factors of ATC patient [17, 24]. However, most of these studies are retrospective and biased and prospective randomized studies are needed in order to confirm such opinions.
